# Djulis Hull Improves Insulin Resistance and Modulates the Gut Microbiota in High-Fat Diet (HFD)-Induced Hyperglycaemia

**DOI:** 10.3390/antiox11010045

**Published:** 2021-12-26

**Authors:** Yu-Tang Tung, Jun-Lan Zeng, Shang-Tse Ho, Jin-Wei Xu, I-Hsuan Lin, Jyh-Horng Wu

**Affiliations:** 1 Graduate Institute of Biotechnology, National Chung Hsing University, Taichung 402, Taiwan; peggytung@nchu.edu.tw; 2 Nutrition Research Center, Taipei Medical University Hospital, Taipei 110, Taiwan; 3 Cell Physiology and Molecular Image Research Center, Wan Fang Hospital, Taipei Medical University, Taipei 116, Taiwan; 4 Department of Forestry, National Chung Hsing University, Taichung 402, Taiwan; arashi0221@smail.nchu.edu.tw (J.-L.Z.); ecsgunro@gmail.com (J.-W.X.); 5 Department of Wood Based Materials and Design, National Chiayi University, Chiayi 600, Taiwan; stho@mail.ncyu.edu.tw; 6 Bioinformatics Core Facility, University of Manchester, Manchester M13 9PT, UK; i-hsuan.lin@manchester.ac.uk; 7 TMU Research Center of Cancer Translational Medicine, Taipei Medical University, Taipei 110, Taiwan

**Keywords:** djulis, hull, type 2 diabetes, antioxidant enzyme, gut microbiota, tight junction protein

## Abstract

In this study, we annotated the major flavonoid glycoside, rutin, of djulis hull crude extract using a Global Natural Products Social Molecular Networking (GNPS) library and its MS/MS spectra. To evaluate the protective effect of djulis hull crude extract and rutin on glucose tolerance, we fed mice a high-fat diet (HFD) for 16 weeks to induce hyperglycaemia. These results showed that crude extract significantly decreased HFD-induced elevation in the area under the curve (AUC) of weekly random blood glucose and oral glucose tolerance tests (OGTT), homeostasis model assessment (HOMA-IR), and advanced glycation end product (AGE) levels, and significantly increased pIRS1 and Glut4 protein expression in epididymal white adipose tissue (eWAT) and liver. Furthermore, the HFD-induced reduction in the activity of glutathione peroxidase (GPx) and catalase (CAT) was reversed by crude extract. In addition, ZO-1 and occludin protein expression in the colon was markedly downregulated in HFD-fed mice, resulting in decreased intestinal permeability and lipopolysaccharide (LPS) translocation, but were restored following crude extract. Moreover, the crude extract intervention had a profound effect on the alpha diversity and microbial community in the gut microbiota. Therefore, djulis hull crude extract could improve blood glucose and increase insulin receptor sensitivity in HFD-induced hyperglycaemia, which is likely due to its modulation of the gut microbiota, preservation of the integrity of the intestinal barrier to reduce body inflammation, increased antioxidant activity, and modulation of insulin signalling.

## 1. Introduction

Obesity-related metabolic diseases, including type 2 diabetes mellitus (T2DM), hyperlipidaemia, and hypertension, are common global health burdens [1]. Diabetes mellitus (DM) is a chronic hyperglycaemia caused by defective insulin secretion, defective insulin action, or both. In T2DM, insulin action and/or insulin secretion are impaired, and impaired insulin secretion is called insulin resistance. In adults, T2DM is the most common type of diabetes, accounting for 90–95% of all diabetic patients [2,3]. Insulin resistance in obese and T2DM patients is manifested by decreased insulin-stimulated glucose transport and metabolism in adipocytes and skeletal muscle and impaired inhibition of hepatic glucose output [4]. By activating the phosphatidylinositol 3-kinase (PI3K)/protein kinase B (AKT) signalling cascade, glucose uptake involving multiple enzymes can reduce glucose levels in the extracellular milieu, thereby contributing to reduced hyperglycaemia. When insulin binds to the insulin receptor (IR) on the surface of target cells, it will cause conformational changes in IR, leading to phosphorylation of insulin receptor (pIR) and phosphorylation of insulin receptor substrate 1 (pIRS-1). Insulin activates the PI3K-AKT pathway, resulting in the transfer of Glut4 from storage vesicles to the plasma membrane and the transport of glucose to skeletal muscle cells [5].

The gut microbiota is an important factor that regulates energy homeostasis and the immune system [6,7]. Regulation of the gut microbiota can improve various diseases, including metabolic syndrome, diabetes, and inflammatory diseases, and influence pharmacotherapy [8,9,10]. Recently, it has been reported that various interventions, including dietary supplementation [11,12], prebiotics or probiotics [13,14], and drugs [15,16], can change the composition of the gut microbiota, which plays an important role in alleviating metabolic diseases.

Djulis (*Chenopodium formosanum*), used as a local cereal, contains starch, dietary fibre, proteins, grain-limited essential amino acids, polyphenols, and phytochemicals [17]. Both dietary fibre and polyphenols may impact the composition of gut microbiota, but, at the same time, the metabolism of polyphenols and their bioavailability depends on the gut microbiota’s composition [18]. Previous studies have shown that djulis has antioxidant, liver protective, skin protective, hypolipidaemic, hypoglycaemic, and antitumour activities [17,19,20,21,22]. Djulis hull is considered agricultural waste that is usually removed during food processing. Although a lot of research has been conducted on the pharmacological activity of djulis, the therapeutic effect of djulis hull and its major compound, rutin, on hyperglycaemia caused by HFD has not been studied yet. Therefore, the aim of the present study was to investigate its effects on obesity, insulin sensitivity, the gut microbiota and the underlying molecular mechanisms in HFD-induced hyperglycaemia.

## 2. Materials and Methods

### 2.1. Extraction, Identification, and Quantitation of the Major Compounds from Djulis Hull

Fresh hulls of djulis were obtained from Sin Fong Farm (Taitung, Taiwan). The hulls were extracted according to our previous report [23]. Djulis hull methanolic crude extracts were analysed using an UHPLC (ultrahigh-performance liquid chromatography; UltiMate 3000 Rapid Separation Dual System, Thermo Fisher Scientific, San Jose, CA, USA) system coupled with a high-resolution mass spectrometer (Orbitrap Fusion Lumos Tribrid mass spectrometer, Thermo Fisher Scientific) in positive ion detection mode. The solid phase was an ACQUITY UPLC BEH C18 column (1.7 µm, 50 × 2.1 mm; Waters, MA, USA). For the mobile phase, the elution conditions were 0–1.0 min of 5% A (acetonitrile + 0.1% formic acid) to B (H_2_O + 0.1% formic acid), 1.0–11.0 min of 5–100% A to B (linear gradient), 11.0–13.0 min of 100% A to B, 13.0–13.2 min of 100–5% A to B (linear gradient), and 13.2–15.0 min of 5% A. The column oven temperature was 40 °C, and the flow rate was 0.4 mL/min. The mass data were converted to mzXML format and uploaded to Global Natural Products Social Molecular Networking (GNPS, https://gnps.ucsd.edu/ProteoSAFe/static/gnps-splash.jsp, accessed on 22 June 2021). The molecular networking analysis was conducted according to the GNPS documentation. The results and original mass data are publicly available at https://gnps.ucsd.edu/ProteoSAFe/status.jsp?task=671b21e5e08340bd9978fc4b1e07c660 (accessed on 22 June 2021) and https://gnps.ucsd.edu/ProteoSAFe/result.jsp?task=39185f888dd64b039b668e8f57642440&view=advanced_view (accessed on 8 August 2021), respectively.

Rutin, the major compound of djulis hull methanolic crude extracts, was further quantified using an HPLC-PDA system (SPD-M20A diode array detector, Shimadzu, Kyoto, Japan) coupled with a C18HQ column (5 µm, 250 × 4.6 mm; Interchim, Montluçon, France). The elution conditions were 0–2.0 min of 4% A (MeOH) to B (H_2_O + 0.05% phosphoric acid), 2.0–42.0 min of 4–100% A to B (linear gradient), 42.0–45.0 min of 100% A to B, 45.0–47.0 min of 100–4% A to B (linear gradient), and 47.0–50.0 min of 4% A. The rutin standard (Sigma-Aldrich Corp. St. Louis, MO, USA) was prepared in various concentrations to establish a calibration curve. Ten microlitres of djulis hull methanolic crude extract (10 mg/mL) was injected into the HPLC system, and the concentration of rutin was calculated according to the calibration curve.

### 2.2. Animals

Male C57BL/6J mice were purchased from the National Laboratory Animal Center (Taiwan) at 7 weeks of age. The mice were housed in a temperature-controlled (22 ± 2 °C) animal centre and a humidity-controlled room with a 12:12-h light–dark cycle (light on at 07:00). Mice were given free access to water and food throughout the experiment. After 1 week of acclimatization, the mice were fed either a normal diet (ND; *n* = 6; Laboratory Rodent Diet 5001, LabDiet, St. Louis, MO, USA; 13% fat with an energy density of 2.89 kcal/g) or a high-fat diet (HFD; *n* = 24; Diet #D12079B, Research Diets, New Brunswick, NJ, USA; 21% fat and 0.15% cholesterol with an energy density of 4.67 kcal/g) for 16 weeks to induce obesity and hyperglycaemia. The HFD-fed mice were then divided into four experimental groups (*n* = 6/group) and treated without (HFD group) or with a dose of 50 mg/kg and 250 mg/kg djulis hull crude extract (HFD/LCE group and HFD/HCE group), and 50 mg/kg rutin (HFD/R group). Crude extract or rutin was dissolved in distilled water and administered by oral gavage once daily for 16 weeks while continuing the HFD. The ND and HFD groups were administered distilled water alone. Body weight, water intake, and diet consumption were measured every week throughout the study. At the end of 16 weeks, animals were fasted for 12 h and euthanized. Blood samples were collected by cardiac puncture. The blood samples were centrifuged at 1500× *g* for 10 min at 4 °C to obtain plasma and serum. The liver, fat around the epididymis, and colon tissues were immediately excised, rinsed, snap-frozen in liquid nitrogen and stored at −80 °C until further analysis. Additionally, the pathological histology of fat around the epididymis was fixed in 4% formaldehyde for a histopathological examination. The experimental design was approved by the Institutional Animal Care and Use Committee of Taipei Medical University (LAC–2020–229) and performed in accordance with the NIH guide for the care and use of laboratory animals.

### 2.3. Blood Biochemical Measurement

Blood samples were obtained from the tail on Weeks 1, 4, 9, 12, and 16, and glucose was measured with a GlucoSure VIVO blood glucose meter (APEXBIO, Hsinchu, Taiwan). The area under the curve (AUC) was calculated from the glucose on Weeks 1, 4, 9, 12 and 16 using GraphPad Prism 6.0 (GraphPad software, San Diego, CA, USA). At the 16th week of the experimental period, plasma insulin levels were measured using Mercodia Mouse Insulin ELISA (Cat# 10-1247-01, Mercodia, Winston Salem, NC, USA), and the homeostasis model assessment (HOMA-IR) was calculated using the following equation: HOMA-IR = (glucose (mg/dL) × insulin (mIU/L))/405.

### 2.4. Oral Glucose Tolerance Test (OGTT)

At 15th week, mice were fasted for 12 h, and an OGTT was performed. OGTT was carried out by administering glucose at a dose of 2.0 g/kg body weight. Blood samples were collected from the tail vein for glucose measurement 0, 30, 60, 90 and 120 min after glucose administration, and blood glucose was measured with a GlucoSure VIVO blood glucose meter. The AUC was derived from the OGTT.

### 2.5. Measurement of Advanced Glycation End Products (AGEs) and Lipopolysaccharide (LPS) Levels

Serum AGEs and LPS levels were measured using the Mouse Advanced Glycation End Products ELISA kit (Cat# CSB-E09414 m, CUSABIO, Houston, TX, USA) and ToxinSensor Chromogenic LAL Endotoxin Assay kit (Cat# L00350, GenScript, Piscataway, NJ, USA), respectively.

### 2.6. Histopathology Analysis

Formaldehyde-fixed and paraffin-embedded epididymal white adipose tissue (eWAT) tissue was sectioned into 4-μm-thick sections, stained with haematoxylin and eosin, and then imaged at 30× magnification. Sections were examined by a clinical pathologist under a light microscope equipped with an Olympus EP50 camera (Olympus, Shinjuku-ku, Tokyo, Japan). One hundred adipocytes were randomly selected from each section, and the areas of individual adipocyte profiles were recorded. Analysis of the adipocyte areas was performed using the ImageJ software package (NIH, Bethesda, MD, USA).

### 2.7. Western Blot Analysis

The protein expression levels of eWAT, liver, and colon were determined using Western blotting following a previously described protocol [24]. The membranes were incubated with primary antibodies (PPAR-γ, pIRS1, IRS1, pAKT, AKT, Glut4, α-tubulin, ZO-1, occludin, Claudin-1, and β-actin) at room temperature for 2 h. In this study, the antibodies were anti-PPAR-γ (Cat# 2435S, 1:1000, Cell Signaling Technology, Danvers, MA, USA), anti-pIRS1 (Ser307) (Cat# AF3272, 1:500, Affinity Biosciences, Cincinnati, OH, USA), anti-IRS1 (Cat# AF6272, 1:500, Affinity Biosciences), anti-pAKT (Cat# AF0016, 1:1000, Affinity Biosciences), anti-AKT (Cat# AF6259, 1:1000, Affinity Biosciences), anti-Glut4 (Cat# AF5386, 1:1000, Affinity Biosciences), anti-α-tubulin (Cat# 2144S, 1:10000, Cell Signaling Technology), anti-ZO-1 (Cat# 21773-1-AP, 1:1000, Proteintech, Rosemont, IL, USA), anti-occludin (Cat# DF7504, 1:1000, Affinity Biosciences), anti-Claudin-1 (Cat# AF0127, 1:1000, Affinity Biosciences), anti-β-actin (Cat# GTX109639, 1:10000, Genetech, South San Francisco, CA, USA), and anti-rabbit IgG (Cat# 2729, 1:1000, Cell Signaling Technology). Immunoreactive bands were visualized by enhanced chemiluminescence ECL for routine immunoblotting. The relative expression of proteins was analysed by densitometry using ImageJ software (Wayne Rasband, Madison, WI, USA). The values were normalized to α-tubulin or β-actin for eWAT, liver, and colon and expressed as the fold increase.

### 2.8. Measurement of Antioxidant Enzyme Activities in the Liver

The antioxidant enzyme activities of glutathione peroxidase (GPx) (Cat#703102, Cayman Chemical Company, Ann Arbor, MI, USA) and catalase (CAT) (Cat#707002, Cayman Chemical Company) were measured using Cayman assay kits. Superoxide dismutase (SOD) (Cat#19160, Sigma–Aldrich, St. Louis, MO, USA) activity was measured using a Sigma–Aldrich assay kit.

### 2.9. Faecal Microbiota Analysis

Amplification and library construction of the 16S rRNA gene were performed using Illumina’s recommended protocols (https://support.illumina.com/downloads/16S_metagenomic_sequencing_library_preparation.html, accessed on 13 October 2020). Briefly, the universal primers 341F (5′-CCTACGGGNGGCWGCAG-3′) and 805R (5′-GACTACHVGGGTATCTAATCC-3′) with Illumina overhang adapter sequences in the forward (5′-TCGTCGGCAGCGTCAGATGTGTATAAGAGACAG-3’) and reverse (5′-GTCTCGTGGGCTCGGAGATGTGTATAAGAGACAG-3’) primers were used to amplify the V3-V4 region of the bacterial 16S rRNA gene by limited cycle PCR. Next, the Nextera XT Index kit (Illumina Inc., San Diego, CA, USA) was used to adapt the Illumina sequencing adapters and dual-index barcodes to the targets in the amplicon, and the quantity and quality of the sequenced library were checked by a QSep100 Analyser (BiOptic Inc., New Taipei, Taiwan). Finally, the libraries were normalized and pooled in an equimolar ratio and sequenced by an Illumina MiSeq instrument using v3 chemistry to generate paired-end reads of 300 bases in length. The universal primer sequences and low-quality reads were trimmed by cutadapt (v1.18) [25], and a DADA2/phyloseq workflow was used to process and analyse the 16S rRNA gene sequence in the R environment. In brief, the filtering, trimming, dereplication, and denoising of the forward and reverse reads were performed using DADA2 (v1.10.1) [26]. The taxonomy of the inferred amplicon sequence variants (ASVs) was assigned by the SILVA reference database (v132) [27] with a minimum bootstrap confidence of 80. Multiple sequence alignment of the ASVs was performed using DECIPHER (v2.10.2) [28]. The frequency table and taxonomy were used to construct a phyloseq object for downstream bacterial community analyses using phyloseq (v1.26.1) [29]. Finally, the R package GUniFrac was used to calculate different versions of the UniFrac distances [30]. The linear discriminant analysis (LDA) effect size (LEfSe) method [31] was used for microbiota enrichment analysis, and GraPhlAn [32] was used to visualize it as a cladogram.

### 2.10. Faecal Short-Chain Fatty Acid Analysis

Faecal short-chain fatty acids (SCFAs) were extracted and analysed according to a previous report [33]. Briefly, faecal samples were suspended in 1 mL of water with 0.5% phosphoric acid and extracted using ethyl acetate. The organic phase was analysed by a GC–MS system consisting of an Agilent 5977B coupled with a 7693A autoinjector (Agilent Technologies, Palo Alto, CA, USA). The identification of SCFAs was based on the retention time of standard compounds (acetic acid, propionic acid, and butyric acid) and the NIST 08 and Wiley7N libraries.

### 2.11. Statistical Analysis

Results were expressed as the mean ± SEM. The significant differences between groups were analysed by one-way ANOVA followed by a post-hoc Duncan’s test; *p* < 0.05 was considered the significance level.

## 3. Results

### 3.1. The Metabolomic Analysis of Djulis Hull Crude Extract

**Figure 1:**
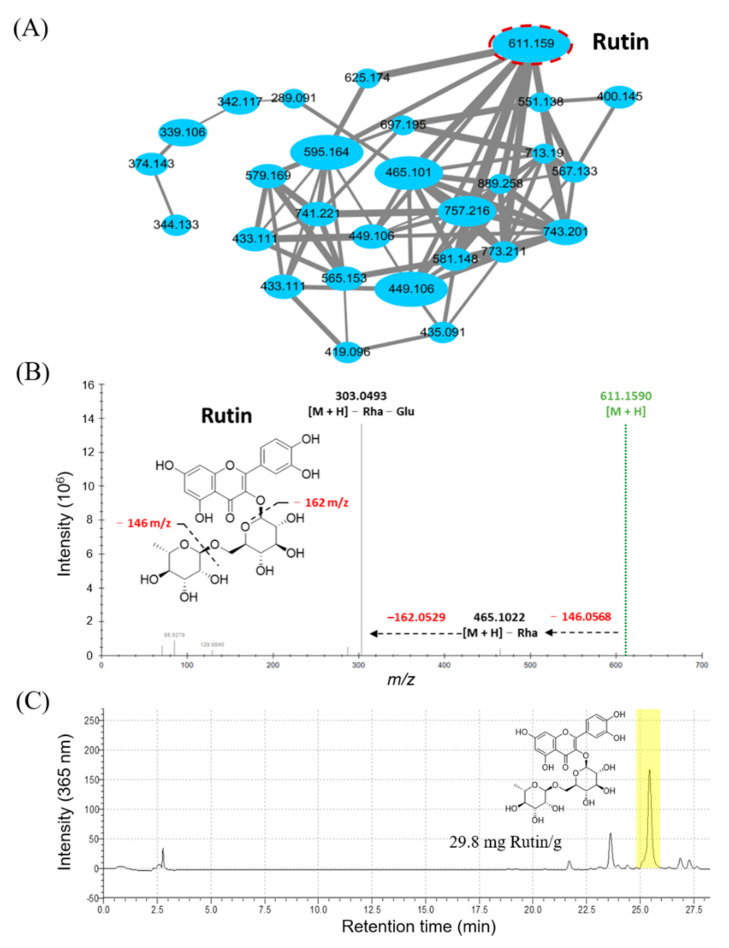
Metabolomic analysis of djulis hull crude extract. (**A**) Flavonoid glycosides-related cluster. (**B**) MS/MS fragmentation of rutin. (**C**) HPLC profiling (365 nm) of djulis hull crude extract.

The molecular networking analysis of djulis hull methanolic crude extracts was conducted using GNPS. Our results revealed that more than 1000 signals (1174 nodes and 68 molecular clusters) were found in the molecular networking analysis. Among those signals, the structures of 93 metabolites were annotated by the GNPS library (https://gnps.ucsd.edu/ProteoSAFe/status.jsp?task=671b21e5e08340bd9978fc4b1e07c660, accessed on 22 June 2021). Flavonoids could improve the pathogenesis of diabetes and its complications through regulation of the glucose metabolism [34]. Based on the results of metabolite structure annotation, a flavonoid glycoside-related cluster (28 nodes) was highlighted (Figure 1A). Among these nodes, *m/z* 611.159 had the highest relative intensity, which was further identified as rutin according to the GNPS library and its MS/MS spectra (Figure 1B). Additionally, the rutin content (29.8 mg/g of djulis hull methanolic crude extract) was estimated in this study (Figure 1C). Therefore, the antihyperglycaemic effects of rutin were further investigated in this study.

### 3.2. Effects of Djulis Hull Crude Extract and Rutin on Morphology, Body Weight, Water Intake, Caloric Intake, and Random Blood Glucose in High Fat Diet-Induced Hyperglycaemia

**Figure 2:**
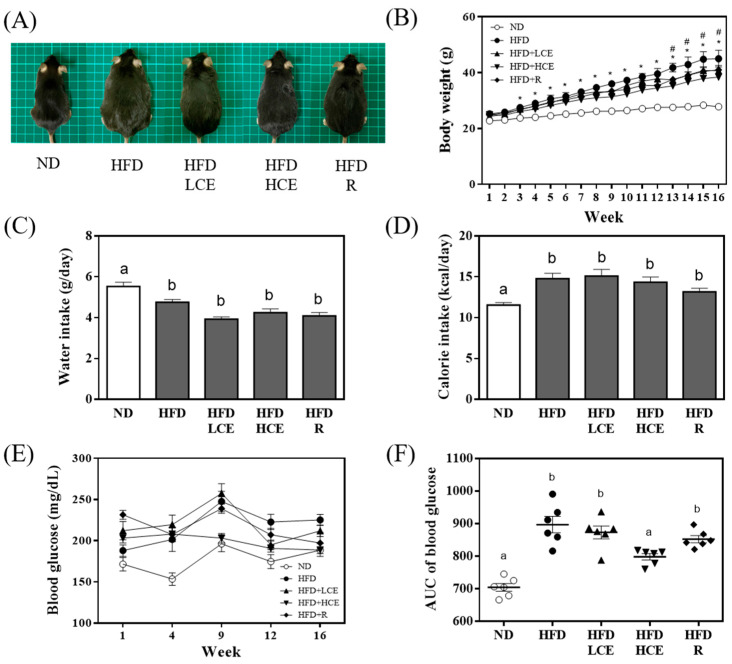
Effect of djulis hull crude extract and rutin on morphology, body weight, water intake, calorie intake, and random blood glucose in high fat diet-induced hyperglycaemia. (**A**) Representative pictures of mice. (**B**) Growth curve of body weight. (**C**) Average water intake. (**D**) Average calorie intake. (**E**) Weekly random blood glucose levels. (**F**) AUC of weekly random blood glucose. ND: normal diet; HFD: high-fat diet; LCE: low dosage of crude extract; HCE: high dosage of crude extract; R: rutin; AUC: area under the curve. Values represent the mean ± SEM (*n* = 6). The statistical methods used one-way ANOVA, and the values with different letters and symbols are significantly different at *p* < 0.05. * *p* < 0.05 ND vs. HFD; ^#^ *p* < 0.05 HFD vs. HFD/HCE.

The effects of djulis hull crude extract and rutin on body weight, water intake, caloric intake, and random blood glucose were evaluated in HFD-induced hyperglycaemia. Mice fed a normal diet gained 9.3 g by the end of the study, whereas those fed the HFD gained an average of 24.4 g (*p* < 0.05). Interestingly, mice fed the HFD containing HCE gained significantly less weight than those consuming only the HFD, particularly after 13 weeks of the study (*p* < 0.05) (Figure 2B). The average water intake was significantly lower in the HFD-fed mice than in the normal diet-fed mice (*p* < 0.05); however, there was no significant difference in the four HFD-fed groups (HFD, HFD/LCE, HFD/HCE, and HFD/R) (Figure 2C). Despite the difference in weight gain between the HFD and HFD/HCE groups, there was no significant difference in caloric intake (Figure 2D). The AUC of weekly random blood glucose in the HFD-fed mice was significantly higher than that in the normal diet-fed mice (*p* < 0.05) (Figure 2F). Moreover, the HFD/HCE group significantly decreased the HFD-induced elevation in the AUC of weekly random blood glucose.

### 3.3. Effects of Djulis Hull Crude Extract and Rutin on Glucose Tolerance and AGEs in HFD-Induced Hyperglycaemia

**Figure 3:**
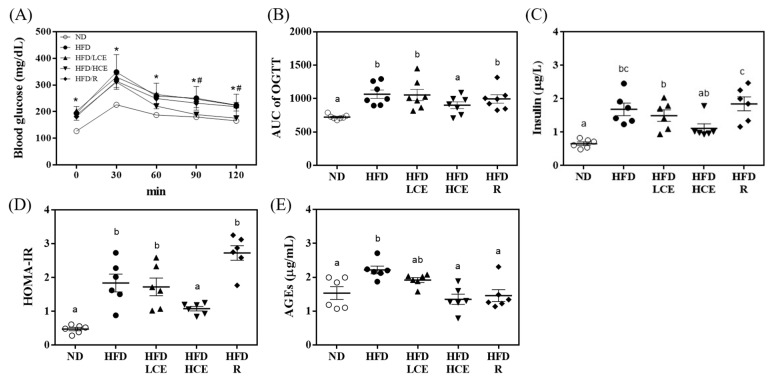
Effects of djulis hull crude extract and rutin on glucose homeostasis and advanced glycation end products (AGEs) in high-fat diet-induced hyperglycaemia. (**A**) Blood glucose was measured every 30 min over 2 h after an oral glucose load. (**B**) AUC of the oral glucose tolerance test (OGTT). (**C**) Insulin. (**D**) Homeostasis model assessment (HOMA-IR). (**E**) AGEs. ND: normal diet; HFD: high-fat diet; LCE: low dosage of crude extract; HCE: high dosage of crude extract; R: rutin; AUC: area under the curve; HOMA-IR = (glucose (mg/dL) × insulin (mIU/L))/405. Values represent the mean ± SEM (*n* = 6). The statistical methods used one-way ANOVA, and the values with different superscript letters are significantly different at *p* < 0.05. * *p* < 0.05 ND vs. HFD; ^#^ *p* < 0.05 HFD vs. HFD/HCE.

As shown in Figure 3A,B, mice fed the HFD had lower glucose tolerance with higher blood glucose levels at each time point after oral glucose intake than the mice fed a normal diet. However, the HFD/HCE group showed significant reductions in glucose levels at 60, 90, and 120 min after oral glucose administration compared with the HFD group (Figure 3A). Accordingly, the AUCs of the OGTT, HOMA-IR, and AGE levels in the HFD group were remarkably greater than those in the ND group (*p* < 0.05). Moreover, the HFD/HCE group significantly decreased the HFD-induced elevation in OGTT, insulin, HOMA-IR, and AGE levels (Figure 3B–E). In addition, the HFD/R group significantly reduced the levels of AGEs but did not affect the levels of OGTT and HOMA-IR. These results demonstrated that the djulis hull crude extract intervention can ameliorate glucose tolerance and AGEs in HFD mice.

### 3.4. Effects of Djulis Hull Crude Extract on the Distribution of Epididymal White Adipose Tissue in HFD-Induced Hyperglycaemia

**Figure 4:**
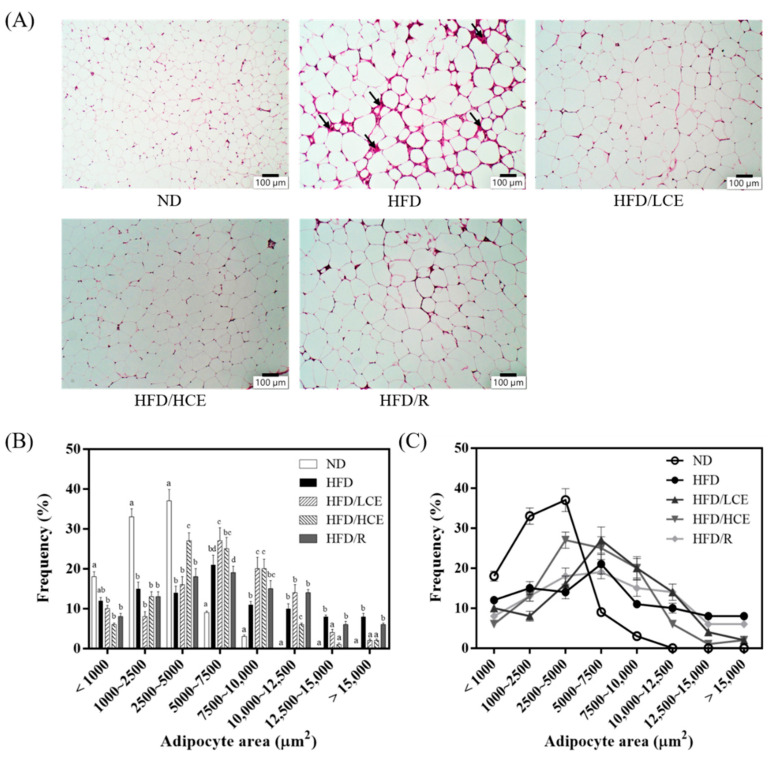
Effect of djulis hull crude extract on haematoxylin & eosin staining and adipocyte distribution of epididymal white adipose tissue (eWAT) in high-fat diet-induced hyperglycaemia. (**A**) Representative haematoxylin and eosin staining. Scale bar: 100 μm. (**B**,**C**) Adipocyte size and distribution of eWAT. ND: normal diet; HFD: high-fat diet; LCE: low dosage of crude extract; HCE: high dosage of crude extract; R: rutin. Values represent the mean ± SEM (*n* = 6). The statistical methods used one-way ANOVA, and the values with different letters are significantly different at *p* < 0.05.

A larger adipocyte size around the epididymis was observed in the HFD group than in the ND group (Figure 4A). HFD-fed mice had an adipocyte distribution shifted to the right, with the highest fraction of large adipocytes (Figure 4B,C). After 16 weeks of the djulis hull crude extract intervention, the HFD/HCE group displayed a significant decrease in adipocyte size, and the adipocyte distribution was shifted to the left, with a notable increase in the fraction of small adipocytes (2500–5000 and 7500–10,000 μm^2^) and a corresponding significant decrease in the fraction of large adipocytes (>10,000 μm^2^) compared with the HFD group. However, the HFD/R group did not change the adipocyte distribution. These results implied that the djulis hull crude extract intervention significantly ameliorated the adverse effect of HFD in causing adipocyte hypertrophy and adipose deposits in the eWAT.

### 3.5. Effects of Djulis Hull Crude Extract on the Expression of Insulin Signalling Proteins in eWAT and the Liver of HFD-Induced Hyperglycaemia

**Figure 5:**
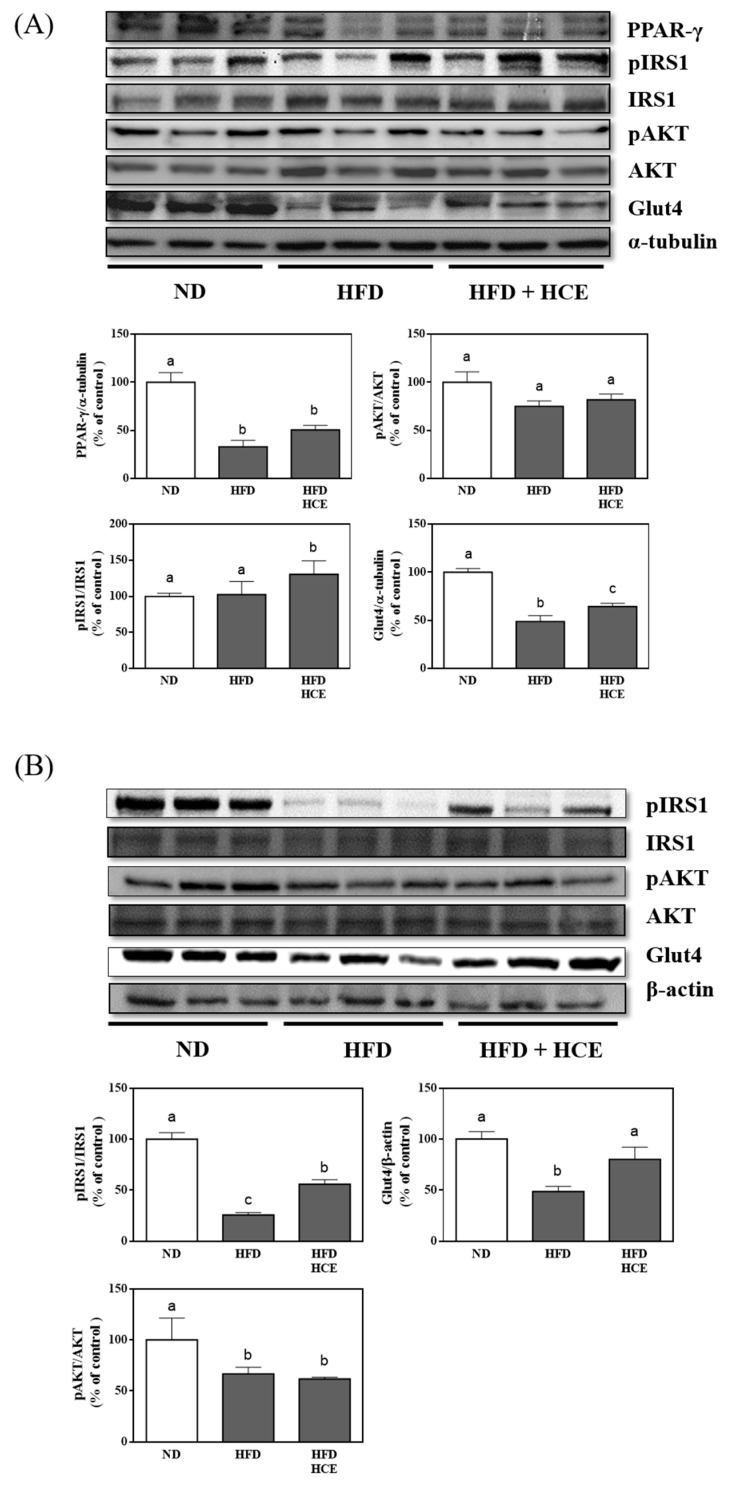
Effect of djulis hull crude extract on the expression of proteins involved in glucose transportation in (**A**) epididymal white adipose tissue (eWAT) and (**B**) the liver of high-fat diet-induced hyperglycaemia. ND: normal diet; HFD: high-fat diet; HCE: high dosage of crude extract. Values represent the mean ± SEM (*n* = 6). The statistical methods used one-way ANOVA, and the values with different letters are significantly different at *p* < 0.05.

To determine the effects of djulis hull crude extract on insulin signalling proteins, we examined the protein expression of PPAR-γ, pIRS1, pAKT, and Glut4 in eWAT (Figure 5A) and liver (Figure 5B). The PPAR-γ and Glut4 protein levels in eWAT of the HFD group were significantly decreased by 67.0% (*p* < 0.05) and 51.2% (*p* < 0.05), respectively, in the HFD group compared with the ND group. However, only Glut4 protein expression was significantly increased in the HFD/HCE group by 31.9% (*p* < 0.05) compared with the HFD group. In addition, no significant difference in the pAKT protein expression of eWAT was observed among the ND, HFD, and HFD/HCE groups. The pIRS1 protein expression in eWAT of the HFD/HCE group was markedly increased compared with that of the ND and HFD groups (*p* < 0.05). The hepatic levels of pIRS1 and Glut4 in the HFD group were significantly decreased compared with those in the ND group (*p* < 0.05). However, the protein expression levels of pIRS1 and Glut4 were significantly elevated in the HFD/HCE group compared with the HFD group (*p* < 0.05). These results implied that the djulis hull crude extract intervention significantly increased pIRS1 and Glut4 in the eWAT and liver.

### 3.6. Effects of Djulis Hull Crude Extract on Antioxidant Enzyme Activities in the Liver under HFD-Induced Hyperglycaemia

**Figure 6:**
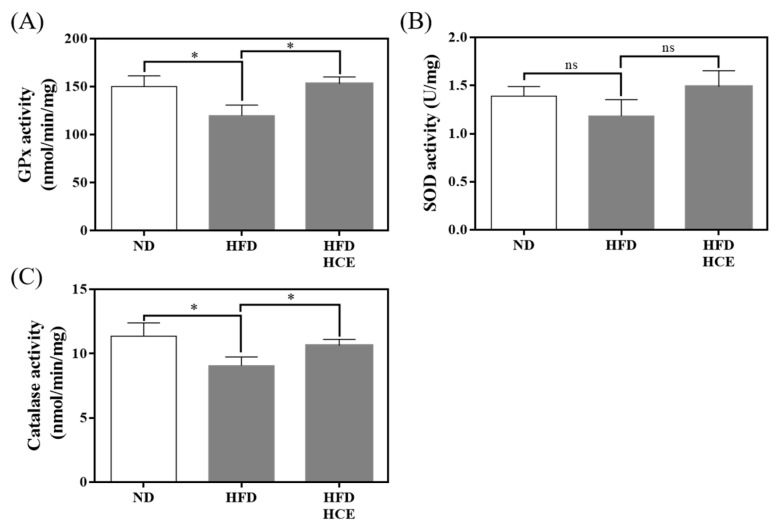
Effect of djulis hull crude extract on the antioxidant enzyme activities of (**A**) glutathione peroxidase (GPx), (**B**) superoxide dismutase (SOD), and (**C**) catalase (CAT) in the liver. ND: normal diet; HFD: high-fat diet; HCE: high dosage of crude extract. Values represent the mean ± SEM (*n* = 6). The statistical methods used an unpaired one-tailed Student’s *t*-test. Significant differences between two different groups are indicated; * *p* < 0.05, ns = not significant.

To determine the effects of djulis hull crude extract on antioxidant enzyme activities, we examined GPx, SOD, and CAT in the liver (Figure 6). The GPx and CAT activities of the HFD group were significantly decreased by 20.1% (*p* < 0.05) and 20.2% (*p* < 0.05), respectively, compared with those of the ND group. However, the HFD/HCE group had significantly elevated GPx and CAT activities by 27.7% (*p* < 0.05) and 17.2% (*p* < 0.05), respectively, compared with the HFD group. In addition, no significant difference in the SOD activity of the liver was observed among the ND, HFD, and HFD/HCE groups. These results implied that djulis hull crude extract intervention significantly increased antioxidant enzyme activities in the liver.

### 3.7. Effects of Djulis Hull Crude Extract on Serum LPS Levels and the Expression of Tight Junction Proteins in the Colon of HFD-Induced Hyperglycaemia

**Figure 7:**
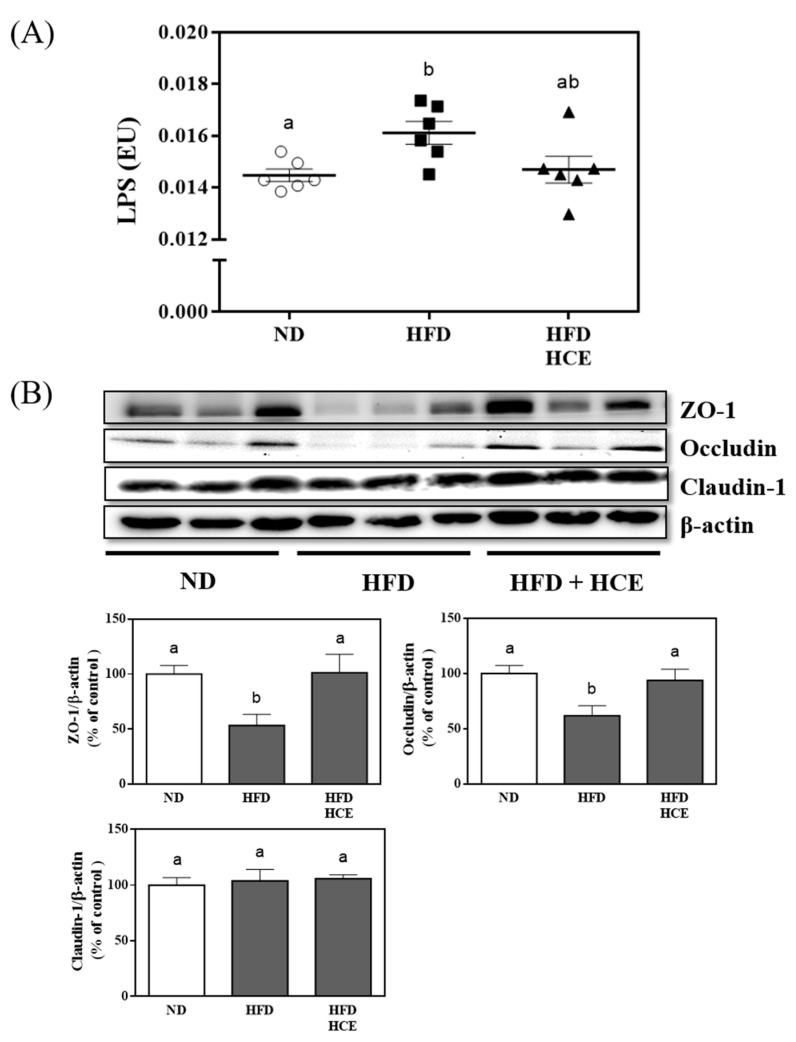
Effect of djulis hull crude extract on (**A**) LPS in serum and (**B**) the protein expression of tight junctions in the colon of high-fat diet-induced hyperglycaemia. ND: normal diet; HFD: high-fat diet; HCE: high dosage of crude extract; LPS: lipopolysaccharide. Values represent the mean ± SEM (*n* = 6). The statistical methods used one-way ANOVA, and the values with different letters are significantly different at *p* < 0.05.

Endotoxin LPS, a cell wall component of almost all Gram-negative bacteria from gut bacteria, is closely related to the development of hyperglycaemia [35]. Thus, we examined the effects of djulis hull crude extract on circulating LPS levels. The serum level of LPS was dramatically increased in HFD-fed mice compared with normal diet-fed mice (*p* < 0.05); however, djulis hull crude extract administration slightly reduced the serum level of LPS (Figure 7A).

Hyperglycaemia increases intestinal permeability, leading to the reduced expression of tight junction proteins [36]. Decreased expression of tight junction proteins leads to increased intestinal permeability and LPS translocation, which plays an important role in the pathophysiology of hyperglycaemia [35,36]. We further evaluated the effects of djulis hull crude extract on intestinal permeability. As shown in Figure 7B, the protein expression levels of ZO-1 and occludin were markedly downregulated in HFD-fed mice (*p* < 0.05) but were restored following djulis hull crude extract administration (*p* < 0.05). These results indicated that the djulis hull crude extract intervention increased tight junction proteins, resulting in decreased intestinal permeability and LPS translocation in HFD-induced hyperglycaemia.

### 3.8. Effects of Djulis Hull Crude Extract on the Gut Microbiota in High-Fat Diet-Induced Hyperglycaemia

**Figure 8:**
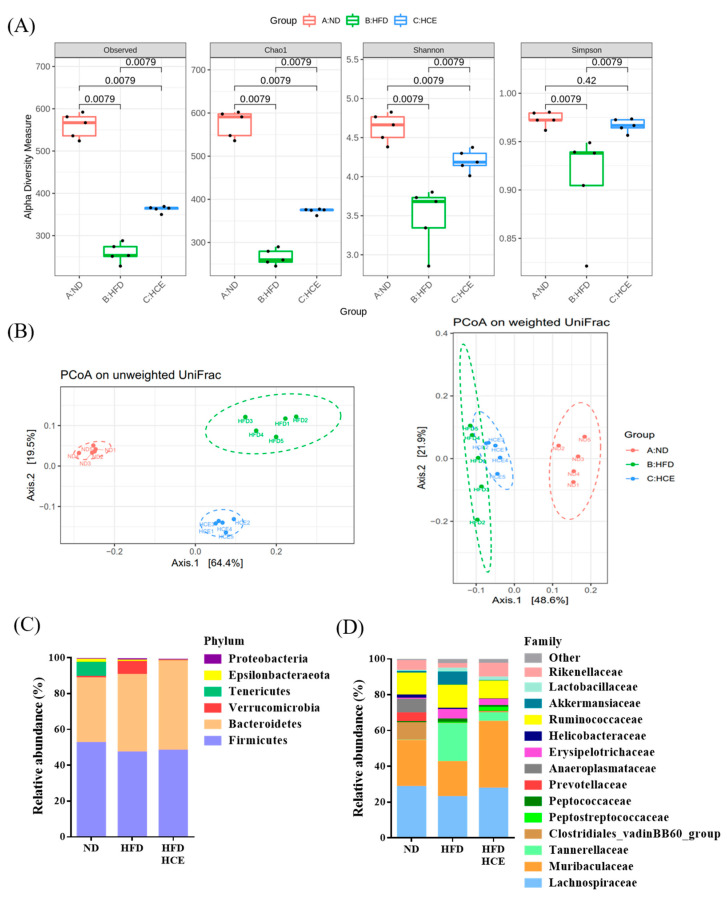
Effect of djulis hull extract on the diversity of the gut microbiota in the faeces of high-fat diet-induced hyperglycaemia. (**A**) Alpha diversity of the observed, Chao1, Shannon, and Simpson indices. (**B**) Beta diversity on unweighted and weighted UniFrac principal coordinate analysis (PCoA) plots. Relative abundance of microbiota species at the (**C**) phylum level and (**D**) family level.

To establish the effects of djulis hull crude extract on gut microbiota composition, we conducted Illumina-generated 16S rRNA amplicon sequencing from 15 samples (*n* = 5 per group). We analysed the gut microbiota composition of the ND, HFD, and HFD/HCE groups in terms of the microbial ecology when treated with a HFD for 16 weeks. Alpha-diversity indices of richness (observed and Chao1) and of richness and evenness (Shannon and Simpson) were significantly decreased in the HFD group compared with the ND group (*p* < 0.05) (Figure 8A). However, a significant increase in the alpha diversity (observed, Chao1, Shannon, and Simpson) of the gut microbiota was observed in the HFD/HCE group compared with the HFD group (*p* < 0.05). Moreover, principal coordinate analysis (PCoA) based on unweighted and weighted UniFrac distances revealed a high degree of divergence between normal diet-treated mice and high-fat diet-treated mice (Figure 8B). Interestingly, the HFD/HCE group grouped the gut microbiota into a different cluster compared with the HFD group, although both of the groups were fed the HFD. These results suggested that diet patterns play a crucial role in modulating gut microbiota composition, and therefore, HFD may induce gut microbiota dysbacteriosis. However, Djulis hull crude extract can improve gut microbiota dysbacteriosis. Figure 8C shows that the overall composition of the gut microbiome in the ND, HFD, and HFD/HCE groups was dominated by the phyla Firmicutes (52.4% for the ND group, 47.1% for the HFD group, and 48.1% for the HFD/HCE group) and Bacteroidetes (36.1% for the ND group, 43.2% for the HFD group, and 49.8% for the HFD/HCE group), in accordance with previous reports of the gut microbiome composition of mice [37,38]. Other phyla detected included Verrucomicrobia, Tenericutes, Epsilonbacteraeota, and Proteobacteria. The HFD group had fewer members of the phyla Firmicutes and Tenericutes and more members of the phyla Bacteroidetes and Verrucomicrobia than the ND group. However, the HFD/HCE group had increased members of the phylum Bacteroidetes and decreased Verrucomicrobia compared with the HFD group. Furthermore, the top 14 families in each group with the highest numbers of assigned sequences with more than 97% of the total sequence are shown in Figure 8D. The HFD group had fewer members of the families Lachnospiraceae, Muribaculaceae, Clostridiales_vadinBB60_group, Prevotellaceae, Anaeroplasmataceae, Helicobacteraceae, and Rikenellaceae and more members of the families Tannerellaceae, Peptostreptococcaceae, Peptococcaceae, Erysipelotrichaceae, Akkermansiaceae, and Lactobacillaceae than the ND group. However, the HFD/HCE group had increased members of the families Lachnospiraceae, Muribaculaceae, Clostridiales_vadinBB60_group, Peptostreptococcaceae, and Rikenellaceae and decreased Tannerellaceae, Peptococcaceae, Erysipelotrichaceae, Helicobacteraceae, Ruminococcaceae, Akkermansiaceae, and Lactobacillaceae compared with the HFD group.

**Figure 9:**
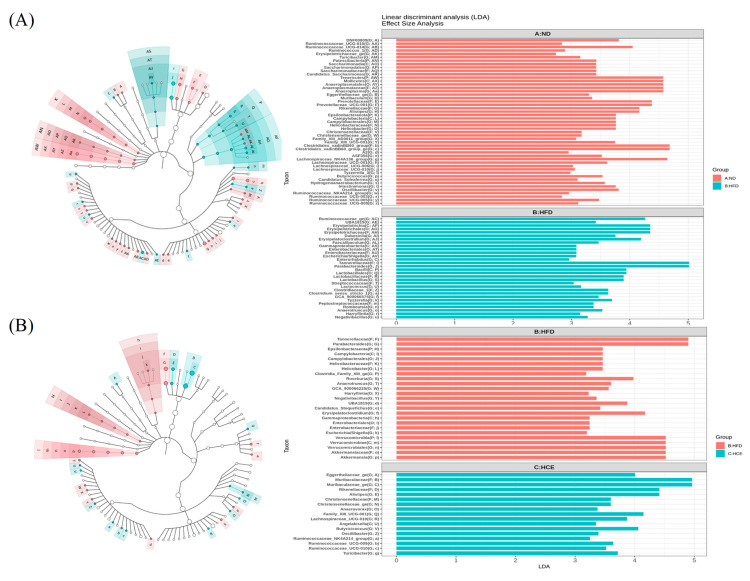

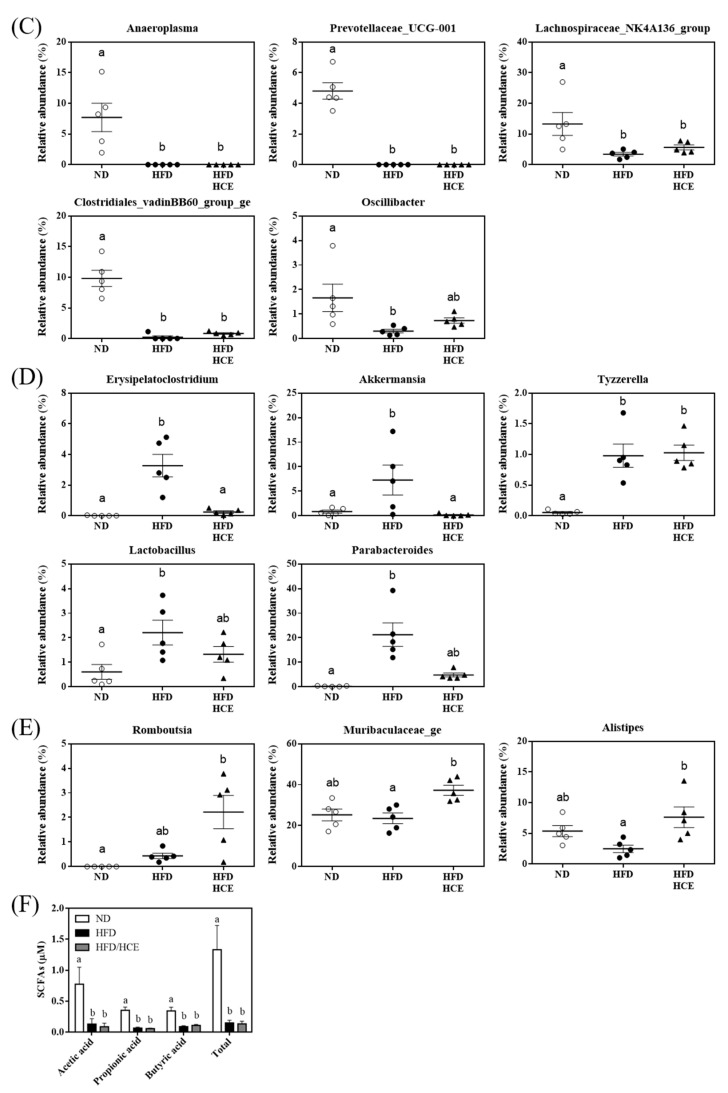
Effects of djulis hull extract on the composition of the gut microbiota in the faeces of high-fat diet-induced hyperglycaemia. Cladogram generated from the linear discriminant effect size (LEfSe) analysis, showing the most differentially abundant taxa enriched in the microbiota of (**A**) ND (red) or HFD (green) and (**B**) HFD (red) or HCE (green). Significantly different abundances in LEfSe comparisons, sorted by *p*-values in ascending order. (**C**) Compared with the ND group, the bacterial genera decreased in the HFD group. (**D**) Compared with the ND group, the bacterial genera increased in the HFD group. (**E**) Compared with the ND or HFD groups, the bacterial genera increased in the HCE group. (**F**) Short-chain fatty acid production in different groups. The statistical methods used one-way ANOVA, and the values with different letters are significantly different at *p* < 0.05.

As shown in Figure 9A, the linear discriminant analysis (LDA) effect size (LEfSe) indicated that the genera *Ruminococcaceae_ge*, *Erysipelatoclostridium*, *Parabacteroides*, *Lactobacillus*, and *Tyzzerella* were higher in HFD-treated mice than in ND-treated mice, while *Ruminococcaceae_UCG-014*, *Anaeroplasma*, *Prevotellaceae_UCG-001*, *Clostridiales_vadinBB60_group_ge*, *Lachnospiraceae_NK4A136_group*, and *Oscillibacter* were lower in HFD-treated mice than in ND-treated mice. Figure 9B shows that the HFD/HCE group had higher *Eggerthellaceae_ge*, *Muribaculaceae_ge*, *Alistipes*, *Family_XIII_UCG-001*, and *Butyricicoccus* than the HFD group. In addition, the HFD/HCE group had lower *Parabacteroides*, *Erysipelatoclostridium*, and *Akkermansia* than the HFD group.

An LDA score greater than 4 was used to depict the gut microbiota. The relative abundance of *Anaeroplasma*, *Prevotellaceae_UCG-001*, *Lachnospiraceae_NK4A136_group*, *Clostridiales_vadinBB60_group_ge*, and *Oscillibacter* was significantly decreased in the HFD group compared with the ND group (*p* < 0.05) (Figure 9C). The HFD/HCE group did not reverse these abundances. The relative abundance of *Erysipelatoclostridium*, *Akkermansia*, *Tyzzerella*, *Lactobacillus*, and *Parabacteroides* was significantly increased in the HFD group compared with the ND group (*p* < 0.05) (Figure 9D). However, the HFD/HCE group had significantly decreased *Erysipelatoclostridium* and *Akkermansia* compared with the HFD group (*p* < 0.05). In addition, the HFD/HCE group had significantly increased *Romboutsia*, *Muribaculaceae_ge*, and *Alistipes* compared with the ND and HFD groups (Figure 9E).

Hyperglycaemia is an inflammatory condition highly associated with the gut microflora and microbial-derived metabolites [39]; thus, we analysed the concentrations of short-chain fatty acids in the faeces (Figure 9F). As expected, the HFD group showed significantly lower faecal acetic acid, propionic acid, butyric acid, and total SCFA concentrations than the ND group. However, HFD/HCE did not increase acetic acid, propionic acid, butyric acid, or total SCFA concentrations compared with the HFD group.

## 4. Discussion

For experimental research on T2DM, a HFD-induced obesity-dependent T2DM mouse model was developed, which reduced insulin sensitivity, resulting in overproduction of glucose [40]. The advantage of the HFD is that it mimics the natural processes and metabolic characteristics of human syndromes while maintaining the treatment response [41]. In our study, mice fed the HFD had a significantly increased AUC of weekly random blood glucose, OGTT, HOMA-IR, and AGE levels, indicating that the HFD promoted insulin resistance. Previous studies pointed out that the HFD (60% fat kcal) has been found to promote insulin resistance [42,43,44,45,46].

Djulis hull crude extract strongly improved T2DM by lowering body weight and blood glucose (Figure 2) during treatment. Insulin resistance is attributable to an imbalance between hepatic glucose production and tissue uptake [47,48]. HOMA-IR measurement has been considered the most practical and accurate approach for insulin sensitivity. In addition to the improvements in blood glucose uptake of djulis hull crude extract (Figure 2), our results also showed that djulis hull crude extract decreased HOMA-IR in HFD-fed mice (Figure 3). Hence, the results implied that the improvement in blood glucose levels may be due to the positive effect of djulis hull crude extract on insulin receptor sensitivity, which provides a new treatment approach for T2DM. In the study, we found that djulis hull crude extract could improve insulin sensitivity, but rutin, the major component, did not improve insulin sensitivity. There are two possible hypotheses. Firstly, rutin and the other components of djulis hull crude extract might have a synergistic/additive effect, making the treatment more effective for improving insulin sensitivity. Secondly, the active component may be the other compounds of djulis hull crude extract, e.g., betanin, quercetin, and 20-hydroxyecdysone [49]. Previous studies showed that betanin, quercetin, and 20-hydroxyecdysone can have beneficial effects on hyperglycaemia in HFD-fed mice [22,50]. When AGEs accumulate in the organism, they will increase the oxidative stress in the body, leading to the risk of diabetes, insulin resistance, cardiovascular disease, kidney damage, and neurodegenerative diseases [51]. The result showed that djulis hull crude extract can reduce the production of AGEs by regulating blood sugar. Rutin can inhibit the production of AGEs, which may be because rutin can act as a carbonyl capture to inhibit the formation of dicarbonyls, the precursor of AGEs [52,53]. Pashikanti et al. [54] also showed that the metabolites of rutin can significantly inhibit the production of glyoxal and methylglyoxal, the precursors of AGEs, thereby reducing the efficacy of AGEs.

Regulation of the gut microbiota has an impact on improvements in obesity and T2DM [55,56]. Recent studies have demonstrated that some pharmacotherapies can improve metabolic diseases by changing the specific composition of the gut microbiota [15,16]. In this study, we found that the number of OTUs in the faeces of mice in the HFD group was lower than that in the faeces of mice in the ND group. In the analysis of alpha diversity results, the observed, Chao1, Shannon, and Simpson indices were significantly increased in the HFD/HCE group compared with the HFD group. Our results showed that djulis hull crude extract had a profound effect on the diversity and microbial community of the gut microbiota in HFD-induced hyperglycaemic mice. According to the results of beta diversity analysis, PCA (principal coordinate analysis) showed that the sample information of the HFD and HFD/HCE groups was relatively concentrated, and there was a certain distance from the ND group. However, the HFD/HCE group was closer to the ND group than the HFD group, indicating that the HFD/HCE group could promote the recovery of gut microbiota in mice fed the HFD. Based on the above analysis, we speculated that the djulis hull crude extract intervention exerts positive effects by regulating the richness, diversity, and microbial community of the gut microbiota in mice with hyperglycaemia induced by HFD.

The phyla Bacteroidetes, Epsilonbacteraeota, Proteobacteria, and Verrucomicrobia are Gram-negative bacteria; Firmicutes and Tenericutes are Gram-positive bacteria. Compared with the ND group, the proportions of Firmicutes, Tenericutes, and Epsilonbacteraeota in the HFD group showed decreasing trends. In addition, Bacteroidetes, Verrucomicrobia, and Proteobacteria showed increasing trends. However, the proportion of Bacteroidetes was increased, and Verrucomicrobia was reduced in the HFD/HCE group compared with the HFD group. Previous studies showed that rats fed a HFD for 8 or 16 weeks had significantly increased Verrucomicrobia phyla [57,58]. In addition, Koliada et al. [59] showed that an increase in Verrucomicrobia is related to obesity. Chang et al. [60] found that the ratio of Firmicutes to Bacteroidetes was significantly increased in a hyperlipidaemia animal model. Contrary to our expectations, the HFD did not cause a significant increase in the ratio of Firmicutes to Bacteroides but rather reduced it. This result is similar to that of a previous study in which long-term consumption of a HFD containing sweetened condensed milk and saturated animal fat in rats for 16 weeks resulted in a decrease in the ratio of Firmicutes to Bacteroidetes [61]. Interestingly, the djulis hull crude extract intervention decreased the ratio of Firmicutes to Bacteroides.

At the genus level, the relative abundance of *Anaeroplasma*, *Prevotellaceae_UCG-001*, *Lachnospiraceae_NK4A136_group*, *Clostridiales_vadinBB60_group_ge*, and *Oscillibacter* significantly decreased in the HFD group compared with the ND group. Our results (e.g., *Anaeroplasma*, *Prevotellaceae_UCG-001*, *Lachnospiraceae_NK4A136_group*, and *Clostridiales_vadinBB60_group*) are consistent with previous research [62,63]. Velázquez et al. [62] observed that HFD-fed mice significantly decreased the abundance of *Anaeroplasma.* Wang et al. [63] showed that treatment with nicotine and HFD significantly decreased the abundance of *Prevotellaceae_UCG-001*. Additionally, the Prevotellaceae and Clostridiaceae families are recognized producers of butyric acid with anti-inflammatory properties [64]. Ma et al. [65] observed that a HFD significantly decreased *Lachnospiraceae_NK4A136_group*, which is a butyrate producer. In addition, Shang et al. [66] showed that fucoidan regulated the gut microbiota and ameliorated intestinal dysbiosis by increasing the abundance of *Clostridiales_vadinBB60_group* in mice with metabolic syndrome. Contrary to our expectations, HFD significantly reduced *Oscillibacter* in the study. Kong et al. [67] showed that HFD-fed mice significantly increased *Oscillibacter*. *Oscillibacter* may mediate HFD-induced gut dysfunction by impairing the integrity of the gut barrier and changing the permeability of the gut, which ultimately leads to gut inflammation [68].

At the genus level, the relative abundances of *Erysipelatoclostridium*, *Akkermansia*, *Tyzzerella*, *Lactobacillus*, and *Parabacteroides* were significantly increased in the HFD group compared with the ND group. However, djulis hull crude extract decreased *Erysipelatoclostridium*, *Akkermansia*, *Lactobacillus*, and *Parabacteroides*. Chitosan oligosaccharides significantly decreased the relative abundance of inflammatory bacteria such as *Tyzzerella* and *Erysipelatoclostridium* [69]. Safari et al. [70] showed that *Lactobacillus*, *Parabacteroides*, *Allobaculum*, and an unknown genus in the Erysipelotrichacease family were boosted by HFD treatment. In contrast, Li et al. [71] observed that the HFD group had a low abundance of *Parabacteroides* in the colon compared with the control group. Song et al. [72] showed that *Lactobacillus* strains can lower cholesterol and improve hyperlipidaemia and hepatic lipid metabolism. *Akkermansia* is a probiotic, and a decrease in *Akkermansia* in the gut is associated with obesity-related metabolic syndrome [73]. In contrast, excessive mucin degradation by *Akkermansia* may lead to inflammatory bowel diseases (IBDs) because the access of luminal antigens to the intestinal immune system is facilitated [74].

At the genus level, the relative abundances of *Romboutsia*, *Muribaculaceae*, and *Alistipes* were significantly increased in the HFD/HCE group compared with the ND or HFD group. Hydroxysafflor yellow A can reduce HFD-induced obesity in C57BL/6J mice by increasing Romboutsia [75]. *Muribaculaceae* can regulate the intestinal pH value, inhibit the proliferation of harmful bacteria, and protect the homeostasis of the intestinal environment [76]. The family Muribaculaceae could produce propionate as a primary fermentation product [77]. Verdam et al. [78] demonstrated that obesity is negatively correlated with the abundance of *Alistipes* in the gut. In contrast, *Alistipes* was positively correlated with body weight, fat mass, serum TC, TG, LEP, IL-6, and LPS contents, as well as PPARγ gene expression [79].

A HFD causes low-grade chronic inflammation of the gastrointestinal tract by changing the gut microbiota and increasing LPS production [80,81]. Germ-free mice fed HFD have less fat accumulation and do not get fatter compared with conventional mice [81]. Therefore, the gut microbiota plays an important role in HFD-induced obesity. In addition, HFD-fed conventional mice have increased TNF-α levels in the blood and ileum, but germ-free mice do not [80,82]. Therefore, a HFD may destroy the composition of the gut microbiota and increase the production of LPS, thereby increasing the entry of LPS into the blood [83]. Patients with IBD have a high incidence of NAFLD (up to 33.6%), even if it is not related to metabolic risk [84], which is closely related to an impaired intestinal barrier. The intestinal barrier protects against the translocation of harmful substances, including bacteria and inflammatory factors [85]. Therefore, interventions can reduce or prevent the progression of NAFLD by maintaining the integrity of the intestinal barrier [86]. In our study, we found that HFD-fed mice had increased LPS levels in the blood. This result is consistent with reports published by previous studies [80,87,88]. Everard et al. [89] reported that the increase in endotoxaemia in HFD-fed mice may be due to the disturbance of tight junction proteins in the jejunum. In this study, we found that a HFD decreased the expression of tight junction proteins, such as ZO-1 and occludin, leading to increased intestinal permeability and LPS translocation. This result is consistent with the reports published by previous studies [80,89]. Thus, HFD-fed mice likely increased blood LPS levels by stimulating LPS production by the gut microbiota and weakening the colonic epithelial barrier, which facilitates LPS egress. However, the djulis hull crude extract intervention could preserve the integrity of the intestinal barrier, which may contribute to regulating blood glucose. The djulis hull crude extract intervention decreased LPS through the gut epithelium to the circulation due to preservation of the integrity of the intestinal barrier and the consequent decrease in intestinal permeability, which improved body inflammation and insulin resistance.

To further examine the mechanism of insulin sensitivity, we analysed the expression of proteins involved in insulin signalling pathways in the liver and adipose tissue. As shown in Figure 5, djulis hull crude extract significantly increased pIRS1 and Glut4 in the liver and adipose tissue, thereby increasing insulin sensitivity. Previous studies pointed that pIRS1 and Glut4 are the indicators of insulin sensitivity [90,91,92,93,94]. Additionally, the high Glut4 level can promote insulin-mediated glucose uptake [95,96,97]. Therefore, the djulis hull crude extract intervention alleviated the decrease in insulin sensitivity induced by HFD by increasing pIRS1 and Glut4.

## 5. Conclusions

The present study demonstrated that the djulis hull crude extract intervention could decrease adipocyte size and improve insulin sensitivity, i.e., the AUC of weekly random blood glucose and OGTT, and HOMA-IR in HFD-induced hyperglycaemia, which is likely due to its modulation of the gut microbiota (an increase in alpha diversity and the microbial community), preservation of the integrity of the intestinal barrier (an increase in ZO-1 and occluding protein expression), mediation of reduced body inflammation (a decrease in LPS), an increase in antioxidant enzymes (an increase in GPx and CAT activities), and modulation of insulin signalling (an increase in pIRS1 and Glut4 protein expression). According to the study, we discovered the new potential therapeutic effect of djulis hull crude extract and, for the first time, we provided a deep analysis of the gut microbiota changes that occurred after djulis hull crude extract supplementation in the context of obesity and diabetes.

## Data Availability

Data are contained within the article.
